# Prevalence and determinants of inadequate dietary diversity among 5–9 years old children in Bangladesh: findings from a nationally representative cross-sectional survey

**DOI:** 10.1136/bmjopen-2026-117343

**Published:** 2026-09-23

**Authors:** Shahnaz Sharmin, Md Mokbul Hossain, Fahmida Akter, Abu Ahmed Shamim, Sakib Rahman, Kaosar Afsana, Dipak Kumar Mitra, Wameq Azfar Raza, Deepika Chaudhery, Malay Kanti Mridha

**Affiliations:** 1 Centre for Non-communicable Diseases and Nutrition, BRAC James P Grant School of Public Health, BRAC University, Dhaka, Bangladesh; 2 BRAC James P Grant School of Public Health, BRAC University, Dhaka, Bangladesh; 3 North-South University, Dhaka, Bangladesh; 4 The World Bank, Dhaka, Bangladesh; 5 The World Bank, New Delhi, Delhi, India

**Keywords:** Cross-Sectional Studies, Child, NUTRITION & DIETETICS, PUBLIC HEALTH, EPIDEMIOLOGY

## Abstract

**Abstract:**

**Objectives:**

To assess the inadequate dietary diversity (IDD) and factors associated with it among 5–9 years old children in Bangladesh.

**Design:**

The study was based on cross-sectional data from the second wave of a longitudinal food security and nutrition surveillance in 2023.

**Setting:**

Data were collected from 90 clusters (64 rural, 16 non-slum urban and 10 slums) in all 8 administrative divisions of Bangladesh.

**Participants:**

A total of 5764 (2850 boys and 2914 girls) children were included in the study. Information was collected from their mothers.

**Outcome measures:**

The primary outcome measure was IDD. Dietary data were collected using a list-based (24 food categories) recall method, which was further grouped into 10 food groups. IDD was defined as consumption of fewer than 5 food groups out of 10.

**Results:**

More than half of the children (52.7%) had IDD (<5 recommended food groups), with slightly higher prevalence among boys (55.0%) than girls (53.2%). Poor parental education (e.g., grade 0–4 (adjusted prevalence ratio (APR): 1.22 for mother, 1.15 for father), grade 5–9 (APR: 1.16 for mother)), unimproved sanitation (APR: 1.07), mild food insecurity (APR: 1.09), severe food insecurity (APR: 1.12) and lower household wealth (APR: 1.19 for the poorest, 1.18 for the poorer), increased the prevalence of IDD. However, children aged 8–9 years (APR: 0.94) and being overweight or obese (APR: 0.82) had a decreased prevalence of IDD compared with their counterparts.

**Conclusions:**

To address IDD among children, multisectoral interventions should be targeted linking improving household wealth and sanitation, reducing food insecurity and advancing parental education.

**STRENGTHS AND LIMITATIONS OF THIS STUDY**

- Our study generates evidence on diet-related data for children aged 5–9 years, as diet-related data for this age group remain insufficient compared with other life phases.
- A nationally representative sample ensures generalisability of the findings for 5–9 years children.
- We used 24-hour dietary recall to obtain dietary data, which might not capture daily variations.
- This study did not explore many other related factors, such as the presence of a home garden, proximity to markets, proximity to health facilities and the contribution of school meals, which could influence the dietary diversity of children.

## Introduction

 Middle childhood (5–9 years) is a period of continued and steady growth that requires good nutrition to support the overall development.^1^ Optimal nutrition is essential for brain and age-appropriate physical and cognitive development in children.^2^ The absence of dietary diversity can impair physical growth and negatively affect children’s cognitive development^3^ and social skills.^4^ A diverse diet is essential to provide sufficient nutrients for good health.^5^

Children in developing countries are particularly vulnerable to nutrition-related health problems, as their diets are largely based on starchy staples, with minimal or no animal source foods and fewer options for vegetables and fruits.^6^ Several studies have demonstrated an association between dietary diversity and nutritional status.^6–8^ For example, a lack of a diversified diet has been identified as a significant indicator of stunting among children under 5 in rural Bangladesh.^8^ Similarly, a study in India indicated a correlation between low dietary diversity scores and underweight in children aged 6–9 years.^9^

Globally, malnutrition remains a concern among 5–9 years old children, with 8.5% being thin, 21.4% overweight and 10.2% obese in 2022.^10^ In Bangladesh, among 5–9 years children, 19.0% were thin, 6.3% were overweight and 2.0% were obese in 2022.^10^ In addition, micronutrient deficiencies, which are often the outcome of low dietary diversity,^11^ are prevalent in Bangladesh. National data show vitamin A deficiency, anaemia prevalence, vitamin D deficiency, iodine deficiency and calcium deficiency accounted for 20.9%, 19.1%, 45.5%, 40.0% and 17.6%, respectively, among 6–14 years old children.^12^

Dietary diversity in children is influenced by multiple, interrelated factors including individual,^13^ parental, household,^14^ cultural^15^ and environmental factors.^16^ 5–9 years of life is crucial to ensure catch-up growth and instil proper eating habits for healthy development.^1^

However, despite its importance, compared with other life phases, this age group has received limited research focus, particularly concerning diet and health.^17^ Diet-related data on middle childhood are insufficient.^1^ In Bangladesh, studies on the dietary diversity of children have mostly covered children under 5 years of age.^18 19^ Until now, there has been little or no information on dietary diversity among 5–9 years old children in Bangladesh using nationally representative data. In this study we aimed to assess the dietary diversity and factors associated with inadequate dietary diversity (IDD) among 5–9 years old children in Bangladesh.

## Methods

### Study design and site

This study used data from the second wave of a longitudinal food security and nutrition surveillance system. The first wave was conducted between 2018 and 2019, and the second wave was carried out from 9 February to 31 October 2023. During the second wave, participants were recruited from all eight administrative divisions of Bangladesh. The survey included seven population groups: children aged 0–4 years, children aged 5–9 years, boys aged 10–19 years, girls aged 10–19 years, women aged 20–59 years, men aged 20–59 years and older adults aged ≥60 years. The surveillance was designed to generate national-level and divisional-level estimates for selected health and nutrition indicators. For the present analysis, we focused only on children aged 5–9 years. Study participants were selected from 90 clusters, including 64 rural clusters, 16 non-slum urban clusters and 10 slum clusters. The study was reported in accordance with the Strengthening the Reporting of Observational Studies in Epidemiology (STROBE) guidelines (online supplemental file 1).

### Sample size and sampling technique

The sample size was determined to generate nationally, and divisionally representative prevalence estimates for the main study variables following the 2018–2019 wave of the Bangladesh Food Security and Nutrition Surveillance. Considering type I error, α=0.05; margin of error, d=0.05; a priori, p=0.5; DEF or design effect=1.61; we estimated 62 children as the required sample size from each cluster.

Different multistage cluster sampling techniques were applied for the selection of the study participants in rural, non-slum urban and slum areas. The selection of rural areas was conducted randomly; first, two districts were selected from each division, followed by the selection of one subdistrict from each selected district. Subsequently, two unions were selected from each subdistrict. Each union was then divided into segments comprising 250–400 households while keeping the geographical boundaries uninterrupted. Finally, two segments were randomly selected from each union, resulting in the selection of 64 rural clusters.

To select the non-slum urban areas, 16 wards were randomly taken, 1–2 wards for each division based on their proportion of urban population. For each ward, Mahallas were picked, and if any of those Mahallas had beyond 500 households, then it was subdivided into smaller sections.

For slum areas, initially, slums containing ≥300 households were selected. Later, slums containing >500 households were identified and segregated into small sections. Finally, as a study cluster, two slums or slum segments from the Dhaka and Chattogram divisions and one slum from the remaining divisions were randomly selected. In total, 90 clusters were selected, with an estimated sample size of 5580 children. The final sample size included in this analysis was 5764 children (2850 boys; 2914 girls).

### Data collection tool and techniques

We collected data through face-to-face interviews with the mothers of children using a structured questionnaire comprising household socioeconomic status, water, sanitation and hygiene, food security, general information about children, dietary diversity and child morbidity. We used TANITA UM-070 digital weighing scale to measure the children’s weight and locally made stadiometers to measure their height. Weight and height were measured at least two times for each child, and a third measurement was taken if the discrepancy between the first two measurements exceeded a certain limit (0.1 kg for weight and 0.5 cm for height). All measurements were performed according to the WHO manual.^20^ Data were captured using tablet computers with the KoboToolBox application.

### Quality control

We field-tested the data collection questionnaire and revised it based on the findings. To ensure the quality of the data, field supervisors re-interviewed about 5% of the previously surveyed households and observed 5% of the interviews. The statistician also generated data queries in case of any discrepancies. The anthropometric instruments were regularly calibrated.

### Outcome variable

We adopted the minimum dietary diversity for women questionnaire to assess dietary diversity in this study. A list-based recall method encompassing 24 food categories consumed in the last 24 hours preceding the interview was used to collect children’s dietary data. Foods were further grouped into ten food groups: (1) grains, white roots, tubers and plantains; (2) pulses (beans, peas and lentils); (3) nuts and seeds; (4) dairy; (5) meat, poultry and fish; (6) eggs; (7) dark green leafy vegetables; (8) other vitamin A-rich fruits and vegetables; (9) other vegetables; and (10) other fruits.^21^ In this study, children consuming 4 or fewer of the 10 food groups in the past 24 hours were considered to have IDD, in line with previous studies.^22 23^

### Explanatory variables

Explanatory variables were chosen based on the existing literature and availability of study data. Table 1 presents the details of the explanatory variables.

**Table 1:** List of the explanatory variables

Variables	Category
Age	5–7 years, 8–9 years
Sex	Male, female
Religion	Islam, others (Hindu, Christian, Buddhist)
Screen time	≤ 2 hours, >2 hours
Any morbidity	No, yes
Nutritional status	Overweight and/or obesity: >+1 SD to >+2 SD Thinness: <−2 SD Normal: >−2 SD and ≤1 SD
Place of residence	Rural, non-slum urban, slum
Maternal education	Grade 0–4, grade 5–9, grade 10 and above
Paternal education	Grade 0–4, grade 5–9, grade 10 and above
Maternal occupation	Homemaker, others
Paternal occupation	Farmer, day labourer, salaried staff, business, others
Household size	1–3 members, 4–5 members, ≥6 members
Access to water	Improved: piped to dwelling, piped to yard/plot, public tap, shared tubewell, household tubewell, covered/protected (dug well), rain water, water tanker, water from the water truck, bottle water Not improved: uncovered/unprotected (dug well), surface water (pond/river/canal/hawar), unprotected (spring)
Access to sanitation	Improved: piped to sewer system, with septic tank, ring slab with water seal, pit latrine with slab Not improved: ring slab without water seal, pit latrine without slab, hanging latrine, no facility (bush/open field/river pond side)
Household food security	Food secure, mild food insecurity, moderate food insecurity, severe food insecurity
Household wealth index	Poorest, poorer, middle, richer, richest

### Data analysis

Stata V.18.5 (StataCorp) was used for data management and analysis. A descriptive analysis was conducted to present the background characteristics of the children and consumption of food groups. The weighted prevalence of IDD was calculated with the 95% CI. Poisson regression with robust variance was carried out to assess the association between the explanatory variables and IDD. In cross-sectional studies involving binomial data, Poisson regression with robust variance may provide more accurate estimates than logistic regression.^24^ The prevalence ratio (PR) is easier to interpret than the OR.^24^ Additionally, OR may overestimate the association compared with PR when the prevalence of an outcome variable exceeds 10%.^25^ For the adjusted model, we considered variables with a p value≤0.2 in the unadjusted analysis.^26^ Variance inflation factor was used to assess multicollinearity among the explanatory variables. Statistical significance was set at p<0.05.

## Results

### Characteristics of the respondents

A total of 5764 children aged 5–9 years were enrolled in this study. Their average age was 7.4 years. Nearly two-thirds (63.1%) of the children were between 5 and 7 years. Approximately half (49.4%) of the children were male, and the majority (85.2%) were from Muslim families. 34% of the children had fever, diarrhoea or acute respiratory illness in the 2 weeks prior to the survey. One-fourth (24.5%) of the children were thin and 7.1% were overweight and obese. A higher proportion (71.6%) of children were from rural areas. Regarding parental educational status, 53.8% of mothers and 42.1% of fathers had studied up to 5–9 grade. Concerning household water and sanitation, 97.8% of the households had access to improved drinking water, while 47.8% had improved sanitation facilities. Over one-third (41.1%) of the households suffered from any form of food insecurity (table 2).

**Table 2:** Background characteristics of the study participants

Variables	n=5764	%
Child characteristics		
Age in years (mean±SD)	7.4±1.4	
Age group (years)		
5–7	3639	63.1
8–9	2125	36.9
Sex of child		
Male	2850	49.4
Female	2914	50.6
Screen time all devices		
≤2 hours	4110	71.3
>2 hours	1651	28.7
Any morbidity (fever, diarrhoea, ARI)		
No	3792	65.8
Yes	1969	34.2
Nutritional status		
Thinness	1401	24.5
Normal	3911	68.4
Overweight or obese	404	7.1
Parental characteristics		
Maternal education		
Grade 0–4	1522	26.4
Grade 5–9	3102	53.8
Grade 10 and above	1140	19.8
Paternal education		
Grade 0–4	2135	37
Grade 5–9	2425	42.1
Grade 10 and above	1204	20.9
Maternal occupation		
Homemaker	4923	85.4
Others	841	14.6
Paternal occupation		
Farmer	604	10.5
Day labourer	1529	26.5
Salaried staff	1036	18.0
Business	1297	22.5
Others	1298	22.5
Household characteristics		
Area of residence		
Rural	4125	71.6
Non-slum urban	1006	17.5
Slum	633	11.0
Household size		
1–3 members	1051	18.2
4–5 members	3310	57.4
≥6 members	1403	24.3
Religion		
Islam	4906	85.2
Other than Islam	855	14.8
Sources of drinking water		
Improved*	5635	97.8
Not improved†	126	2.2
Sanitation facility		
Improved‡	4128	47.8
Not improved§	1633	52.2
Food security		
Food secure	3390	58.8
Mild food insecurity	1356	23.5
Moderate food insecurity	393	6.8
Severe food insecurity	622	10.8
Household wealth		
Poorest	1153	20.0
Poorer	1152	20.0
Middle	1152	20.0
Richer	1152	20.0
Richest	1152	20.0

* Piped to dwelling, piped to yard/plot, public tap, shared tubewell, household tubewell, covered/protected (dug well), rain water, water tanker, water from the water truck, bottle water.

† Uncovered/unprotected (dug well), surface water (pond/river/canal/hawar), unprotected (spring).

‡ Piped to sewer system, with septic tank, ring slab with water seal, pit latrine with slab.

§ Ring slab without water seal, pit latrine without slab, hanging latrine, no facility (bush/open field/river pond side).

ARI, acute respiratory illness.

### Consumption of foods from different food groups

The most commonly consumed food groups were grains, roots and tubers (99.9%), followed by meat, fish or poultry (71.1%), other vegetables (61.6%), other fruits (49.6%) and pulses (46.2%). A small proportion of the children consumed eggs (34.2%), dairy products (31.6%), vitamin A-rich fruits and vegetables (27.6%), green leafy vegetables (26.9%) and nuts and seeds (6%) (figure 1).

**Figure 1:**
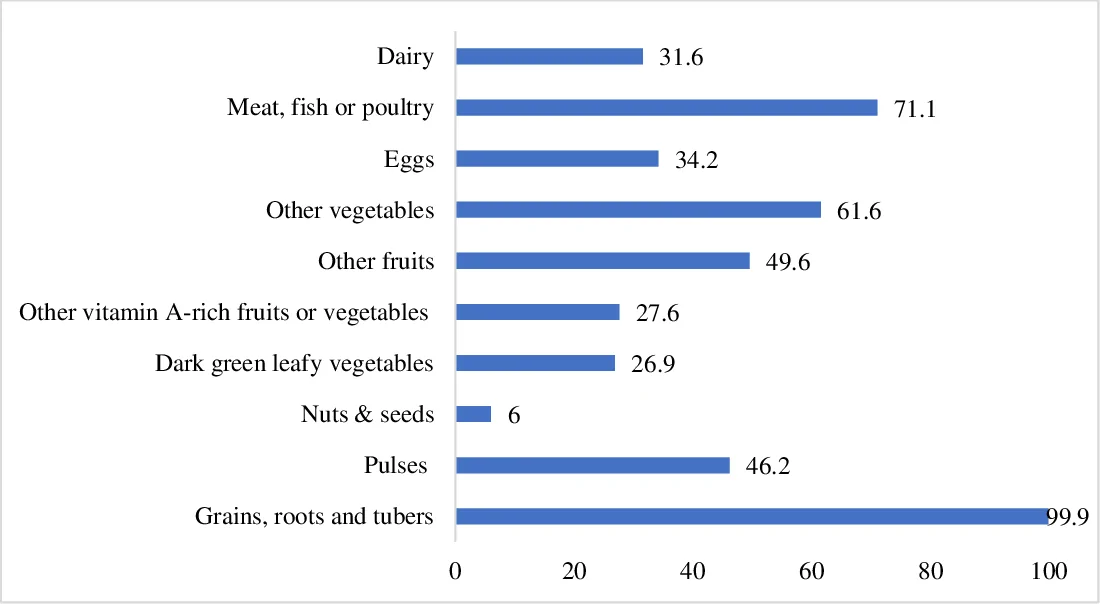
Proportion of children aged 5–9 years by consumption of food from different food groups.

### Prevalence of IDD

The weighted prevalence of IDD among 5–9 years old children was 52.7%, and the prevalence was slightly higher in boys (55.0%) than in girls (53.2%). Compared with mothers and fathers with an educational level of grade 10 or above, children of mothers and fathers with an educational level of grade 0–4 had a higher prevalence of IDD (mothers: 58.8% vs 43.1%; fathers: 59.6% vs 41.6%). The prevalence of IDD was higher among children from food-insecure households than among those from food-secure households. In addition, children from the poorest and poorer households had a higher prevalence of IDD compared with children from the richest households (table 3).

**Table 3:** Prevalence of IDD among 5–9 years children by background characteristics

Variables	IDD% (95% CI)
Overall	52.7 (49.4 to 56.1)
Child characteristics	
Age group (years)	
5–7	54.9 (49.3 to 60.4)
8–9	52.7 (49.3 to 56.0)
Sex of child	
Male	55.0 (51.2 to 58.8)
Female	53.2 (48.2 to 58.1)
Screen time all devices (hours)	
≤2	55.4 (50.3 to 60.4)
>2	50.1 (46.4 to 53.8)
Any morbidity (fever, diarrhoea, ARI)	
No	54.9 (49.5 to 60.3)
Yes	52.4 (47.9 to 56.8)
Nutritional status	
Thinness	54.8 (47.4 to 62.0)
Normal	54.3 (50.2 to 58.4)
Overweight and/or obese	43.8 (35.0 to 53.0)
Parental characteristics	
Maternal education	
Grade 0–4	58.8 (50.8 to 66.5)
Grade 5–9	55.2 (50.9 to 59.4)
Grade 10 and above	43.1 (38.1 to 48.3)
Paternal education	
Grade 0–4	59.6 (53.8 to 65.1)
Grade 5–9	53.6 (49.0 to 58.1)
Grade 10 and above	41.6 (35.7 to 47.8)
Maternal occupation	
Homemaker	54.8 (50.0 to 59.5)
Others	49.1 (40.5 to 57.9)
Paternal occupation	
Farmer	49.5 (39.7 to 59.3)
Day labourer	56.6 (51.2 to 61.9)
Salaried staff	44.9 (37.7 to 52.3)
Business	52.6 (47.1 to 58.0)
Others	59.5 (52.3 to 66.3)
Household characteristics	
Area of residence	
Rural	54.1 (49.8 to 58.3)
Non-slum urban	55.7 (41.8 to 68.7)
Slum	53.0 (41.7 to 64.0)
Household size	
1–3 members	57.0 (51.1 to 62.8)
4–5 members	54.9 (51.1 to 58.7)
≥6 members	51.2 (44.3 to 58.0)
Religion	
Islam	54.8 (50.3 to 59.3)
Other than Islam	48.6 (41.7 to 55.5)
Sources of drinking water	
Improved*	54.1 (49.9 to 58.3)
Not improved†	49.7 (49.4 to 50.0)
Sanitation facility	
Improved‡	51.2 (46.1 to 56.4)
Not improved§	59.2 (53.2 to 64.9)
Food security	
Food secure	51.3 (46.6 to 56.0)
Mild food insecurity	58.4 (51.5 to 65.0)
Moderate food insecurity	60.9 (51.0 to 70.0)
Severe food insecurity	58.6 (51.5 to 65.3)
Household wealth	
Poorest	64.0 (58.8 to 68.8)
Poorer	63.2 (57.2 to 68.8)
Middle	49.0 (41.6 to 56.5)
Richer	42.7 (33.9 to 51.9)
Richest	42.5 (37.8 to 47.4)

* Piped to dwelling, piped to yard/plot, public tap, shared tubewell, household tubewell, covered/protected (dug well), rain water, water tanker, water from the water truck, bottle water.

† Uncovered/unprotected (dug well), surface water (pond/river/canal/*hawar*), unprotected (spring).

‡ Piped to sewer system, with septic tank, ring slab with water seal, pit latrine with slab.

§ Ring slab without water seal, pit latrine without slab, hanging latrine, no facility (bush/open field/river pond side).

ARI, acute respiratory illness; IDD, inadequate dietary diversity.

### Factors associated with IDD

Children aged 8–9 years had a 6.0% lower chance of developing an IDD. In addition, the prevalence of IDD is 18% lower in overweight and obese children.

Regarding parental characteristics, the adjusted prevalence ratio (APR) of IDD was 1.22 (APR: 1.22; 95% CI 1.10 to 1.35) and 1.16 (APR: 1.16; 95% CI 1.06 to 1.27) among children having mothers with an education level below grade 5 and grades 5–9, respectively, compared with those having mothers with an education level of grade 10 and above. In addition, for children whose fathers had an education of grade 0–4, the APR was 1.15 (APR: 1.15; 95% CI 1.04 to 1.26) compared to those whose paternal educational level was grade 10 and above.

In terms of household characteristics, the prevalence of IDD was 7.0% higher among children in households with unimproved sanitation compared with those with improved sanitation. The APR of IDD among children of mild food-insecure households was 9.0% higher (APR: 1.09; 95% CI 1.02 to 1.16) than those of food-secure households. Likewise, the prevalence of IDD was 12% higher among children from severe food insecure households (APR: 1.12; 95% CI 1.03 to 1.21). In addition, the APR of IDD was 19% (APR: 1.19; 95% CI 1.09 to 1.30) higher among children from the poorest households than those from the richest households. The APR was 18% (APR: 1.18; 95% CI 1.08 to 1.29) higher among children from poorer households than children from the richest households (table 4).

**Table 4:** CPR and APR of the factors associated with IDD among 5–9 years children in Bangladesh

Variables	CPR (95% CI)	P value	APR (95% CI)	P value
Child characteristics				
Age group (years)				
5–7	Ref		Ref	
8–9	0.95 (0.90 to 1.00)	0.059	0.94 (0.89 to 0.99)	0.021
Screen time all devices				
≤2 hours	Ref		Ref	
>2 hours	0.88 (0.83 to 0.94)	<0.001	0.96 (0.9 to 1.02)	0.185
Any morbidity (fever, diarrhoea, ARI)				
Yes	Ref			
No	0.99 (0.93 to 1.04)	0.590		
Nutritional status				
Normal	Ref		Ref	
Thinness	1.04 (0.98 to 1.10)	0.160	1.03 (0.97 to 1.09)	0.393
Overweight and/or obese	0.71 (0.62 to 0.81)	<0.001	0.82 (0.72 to 0.94)	0.003
Parental characteristics				
Maternal education				
Grade 0–4	1.52 (1.39 to 1.65)	<0.001	1.22 (1.10 to 1.35)	<0.001
Grade 5–9	1.35 (1.25 to 1.47)	<0.001	1.16 (1.06 to 1.27)	0.002
Grade 10 and above	Ref		Ref	
Paternal education				
Grade 0–4	1.47 (1.36 to 1.59)	<0.001	1.15 (1.04 to 1.26)	0.005
Grade 5–9	1.29 (1.19 to 1.40)	<0.001	1.09 (0.99 to 1.19)	0.068
Grade 10 and above	Ref		Ref	
Household characteristics				
Area of residence				
Rural	Ref		Ref	
Non-slum urban	0.79 (0.73 to 0.85)	<0.001	0.97 (0.89 to 1.06)	0.516
Slum	1.05 (0.97 to 1.13)	0.240	1.07 (0.98 to 1.16)	0.131
Household size				
1–3 members	1.01 (0.93 to 1.10)	0.793		
4–5 members	1.03 (0.97 to 1.10)	0.292		
≥6 members	Ref			
Religion				
Other than Islam	Ref		Ref	
Islam	1.06 (0.98 to 1.14)	0.149	1.07 (0.99 to 1.15)	0.08
Sources of drinking water				
Improved*	Ref			
Not improved†	1.08 (0.92 to 1.27)	0.335		
Sanitation facility				
Improved‡	Ref		Ref	
Not improved§	1.21 (1.15 to 1.28)	<0.001	1.07 (1.01 to 1.13)	0.031
Food security				
Food secure	Ref			
Mild food insecurity	1.20 (1.13 to 1.27)	<0.001	1.09 (1.02 to 1.16)	0.008
Moderate food insecurity	1.19 (1.09 to 1.31)	<0.001	1.07 (0.97 to 1.18)	0.172
Severe food insecurity	1.25 (1.16 to 1.35)	<0.001	1.12 (1.03 to 1.21)	0.006
Household wealth				
Poorest	1.38 (1.27 to 1.49)	<0.001	1.19 (1.09 to 1.30)	<0.001
Poorer	1.33 (1.23 to 1.45)	<0.001	1.18 (1.08 to 1.29)	<0.001
Middle	1.18 (1.08 to 1.29)	<0.001	1.07 (0.98 to 1.17)	0.157
Richer	1.02 (0.93 to 1.12)	0.614	0.99 (0.90 to 1.09)	0.815
Richest	Ref		Ref	

* Piped to dwelling, piped to yard/plot, public tap, shared tubewell, household tubewell, covered/protected (dug well), rain water, water tanker, water from the water truck, bottle water.

† Uncovered/unprotected (dug well), surface water (pond/river/canal/hawar), unprotected (spring).

‡ Piped to sewer system, with septic tank, ring slab with water seal, pit latrine with slab.

§ Ring slab without water seal, pit latrine without slab, hanging latrine, no facility (bush/open field/river pond side).

APR, adjusted prevalence ratios; ARI, acute respiratory illness; CPR, crude prevalence ratios; IDD, inadequate dietary diversity.

## Discussion

In this study, we used nationally representative data to assess the dietary diversity and factors associated with IDD among Bangladeshi children aged 5–9 years. We found that the diet of children consisted of grains, roots, tubers, meat, fish or poultry, with insufficient intake of nuts and seeds, dark green leafy vegetables and vitamin A-rich fruits and vegetables. We also found that 5 out of 10 children in Bangladesh had a less diverse diet. Parental education (mother: grade 0–4, 5–9; father: grade 0–4), unimproved sanitation, household food insecurity (from mild and severe insecure households) and poor household wealth (belonging to the poorest and poorer households) increased the prevalence of IDD. However, children aged 8–9 years and being overweight or obese have a decreased prevalence of IDD.

Unsurprisingly, almost all children consumed carbohydrate-rich foods (grains, roots and tubers), and a higher proportion consumed animal-source foods (meat, fish or poultry), particularly fish. However, nuts and seeds, dark green leafy vegetables, vitamin A-rich fruits and vegetables, dairy and eggs were limited in their diets. Previous research in Bangladesh involving children aged 24–59 months reported that all children consumed rice and a vast majority consumed fish during the 7 days preceding the interview.^8^ Another study carried out among preschool children (1–5 years) in India pointed out that children’s diet was primarily cereal-based with minimal consumption of green leafy vegetables and fruits, dairy products and flesh foods.^27^ In Pakistan, among schoolchildren in Sindh enrolled in classes 2–5, nuts, seeds and eggs were often omitted from their diets.^28^ In 2022, Sagbo and Kpodji found that carbohydrate food (cereal) was consumed by all children (8–17 years) in southern Benin, which is comparable to our findings, although the consumption frequency of dark green vegetables and vitamin A-rich fruits and vegetables was higher than that in our study.^14^ These differences may be explained by geographical location and seasonal variations in the study area.^29^

In our study, over half of the children had IDD, which is consistent with a study conducted on school-aged children (6–12 years) in southern Ethiopia, which reported an IDD prevalence of 58.3%. However, in that study, IDD was defined as the consumption of fewer than five out of nine food groups.^30^ Similarly, another study from southern Ethiopia, which also focused on children aged 6–12, reported an IDD prevalence of (59.4%). This is identical to our findings, although IDD was defined as consuming less than four out of seven food groups.^31^ The prevalence of IDD in our study was higher than that reported in a study conducted in southern Benin, where 25.8% of children and adolescents had IDD.^14^ The variation in the prevalence of IDD could be due to the different numbers of food groups and cut-offs used to define it, as well as geographical, economic and socio-cultural differences.^13^

We found that children with poor parental education were more likely to have IDD, which concurs with previous studies carried out in Bangladesh, Vietnam and Ethiopia among 6–23 months old children.^32 33^ In addition, a study among children (6–59 months) in rural Bangladesh revealed that higher maternal education is associated with a higher dietary diversity score.^18^ Parents’ educational level influences their nutritional knowledge, which in turn affects their children’s diet.^34 35^

Our findings revealed that unimproved sanitation was a strong predictor of IDD in children aged 5–9 years old children. Using data from South Africa, Motadi *et al*^36^ reported that low dietary diversity was more prevalent among children aged 3–4 years who used unimproved toilets (bush and pit toilets) than among those who used flushing toilets.^36^ A prior study conducted in Uganda by Rukundo *et al*^37^ observed higher dietary diversity scores in households that owned toilets.^37^ However, using a large sample of 18 463 children aged 0–23.9 months in India, it was found that a diversified diet and improved sanitation were correlated with better nutritional outcomes.^38^

Children from mild and severe food insecure households were more likely to have dietary inadequacy than children from food secure households. Studies carried out in Bangladesh, including children aged 6–59 months and adolescents aged 10–19 years, and Ghana among children aged 6–12 years, reported a significant association between food-insecure households and low dietary diversity.^18 22 39^ Research indicates that household dietary diversity is an indicator of household food security, and that a correlation exists between individual dietary diversity and household food security.^40 41^

The association between poor household wealth and IDD among children in the current study is akin to a review by Singh and Sharma, who reported a positive correlation between household socioeconomic status and dietary diversity among schoolchildren.^42^ Similarly, another study by Akter *et al* found poor household wealth to be a determinant of IDD among adolescents aged 10–19 years.^22^ A recent study indicated that school-age children and adolescents from medium and high socio-economic status had a lower chance of IDD compared with their counterparts from low socio-economic status.^14^ In Bangladesh, income significantly influences household dietary diversity^43^ (Rashid *et al*). Poorer households focus on meeting their fundamental energy needs through staple foods within their budgets to alleviate hunger.^44^

In this study, we found that children aged 8–9 years had a lower prevalence of IDD than those aged 5–7 years. This finding is consistent with a study from Ethiopia that reported younger children had lower dietary diversity than older children.^13^ This might be due to, as children grow older, their exposure to diverse social environments increases, influencing their learning and food choices. According to this study, overweight and/or obese children had a lower prevalence of IDD. Our finding is inconsistent with a study conducted in China among 8500 schoolchildren, which showed that higher dietary diversity lowers the risk of overweight or obesity.^45^ However, research has shown mixed results regarding dietary diversity and being overweight or obese. Another study conducted among Chinese children revealed that dietary diversity was not associated with overweight or obesity^46^ (Zhao *et al*). Based on preliminary research findings, an increase in dietary diversity is associated with better micronutrient status in older children, although it poses a risk of obesity.^46^

In our work, we did not observe any link between child morbidity, such as having had diarrhoea in the past week, and poor dietary diversity, which contrasts with findings from a previous study conducted among rural children under 5 in Bangladesh.^8^ However, a study on caregivers’ feeding perceptions and practices during episodes of diarrhoea among children aged 0–36 months revealed that caregivers often perceived feeding as harmful during this period and frequently withheld meat, eggs and milk; some also restricted vegetables and fruits.^47^

The study had key strengths, such as a nationally representative sample that ensured generalisability for 5–9 years children. To our knowledge, this is the first study in Bangladesh to assess the dietary diversity and factors associated with IDD among children aged 5–9 years. Nonetheless, this study had some limitations. We used 24 hours list based dietary recall method to obtain dietary data; in contrast to the open recall method, which often leads to socially desirable bias such as exaggerated reporting of high-status food consumption. Besides, 24 hours dietary recall may not capture daily variations and usual dietary habits. We also did not collect information on school-meal exposure; therefore, the potential contribution of school feeding programmes to children’s dietary diversity could not be assessed. Moreover, as the study is cross-sectional, the causal link between IDD and associated factors could not be established and the associations need to be interpreted with caution.

## Conclusions

This study revealed that more than half of the children in Bangladesh consumed an inadequately diversified diet. In addition, we identified association of several household factors such as lower educational level of parents, food insecurity and poor household status with IDD. These findings suggest that programmes aiming to improve dietary diversity among school-aged children should address household-level socioeconomic barriers, food insecurity and parental nutrition awareness. School-based nutrition platforms, including well-designed school meal programmes with diverse foods, may also be considered as a complementary strategy to improve dietary diversity among school aged children, especially in food-insecure and poverty-prone areas.^48^ Future research should include additional household, school and food-environment variables to better understand the drivers of dietary diversity among children in this age group.

## Supplementary material

10.1136/bmjopen-2026-117343 online supplemental file 1

## Data Availability

Data are available upon reasonable request.
