# Supplementary material for: Prevalence and determinants of inadequate dietary diversity among 5–9 years old children in Bangladesh: findings from a nationally representative cross-sectional survey
Source: BMJ Open. 2026 Sep 23;16(9):e117343. doi: 10.1136/bmjopen-2026-117343 (PMC13629976; doi:10.1136/bmjopen-2026-117343)
Supplement: Supplementary file 1 [file bmjopen-2026-117343.reviewer_comments.pdf]

PEER REVIEW HISTORY

BMJ Open publishes all reviews undertaken for accepted manuscripts. Reviewers are asked to complete a checklist review form and are provided with free text boxes to elaborate on their assessment. These free text comments are reproduced below.

ARTICLE DETAILS

Title (Provisional)

Prevalence and determinants of inadequate dietary diversity among 5-9 years old children in Bangladesh: findings from a nationally representative cross-sectional survey

Authors

Sharmin, Shahnaz; Hossain, Md. Mokbul; Akter, Fahmida; Shamim, Abu Ahmed; Rahman, Sakib; Afsana, Kaosar; Mitra, Dipak Kumar; Raza, Wameq Azfar; Chaudhery, Deepika; Mridha, Malay Kanti

---

VERSION 1 - REVIEW

**Reviewer**	**1**
**Name**	**Thomas, Roger**
**Affiliation**	**University of Calgary, Family Medicine**
**Date**	**26-Feb-2026**
**COI**	**None**

This is an excellent well designed, executed and analysed study, with excellent references to comparable studies in other similar countries.

I noted your replicated about 5% of the interviews. Did you discuss and receive feedback from the interviewers on how thoroughly mothers comprehended the questions and the % where the answers were imprecise enough that no response could be recorded.

Could you please summarise your recommendations for specific quantities or weights of foods that need increase in the childrens' diets and enclose your recommendations in a box to highlight them. Also can you provide the costs/pound weight of food needing increases so that the feasibility compared to household income can be assessed.

**Reviewer**	**2**
**Name**	**Nakamura, Tomiyo**
**Affiliation**	**Ryukoku University, Research Institute for Food and Agriculture**

**Date**	**15-Apr-2026**
**COI**	**None**

The aim of this study was to assess the inadequate dietary diversity (IDD) and factors associated with it among 5-9 years old children in Bangladesh. It is very important to address IDD among children. To help improve the manuscript, please consider the following suggestions:

1.The authors mentioned that the weighted prevalence of IDD among 5-9 years old children was 52.7%, and the prevalence was higher in boys (55.0%) than in girls (53.2%). However, these differences are small, at only 2.3%, and have not been adjusted for other factors. Therefore, please soften this expression somewhat.

2.In Bangladesh, school meals primarily in public primary schools in impoverished areas, are provided with the support of NGOs and JICA. These meals consist of a curry-flavored porridge called "khichri," made with soybeans, vegetables, and rice, as well as nutritious bread. If children ate these meals, it would impact IDD. If possible, please provide information on whether any areas provide these school meals. Also, please discuss this in discussion part.

VERSION 1 - AUTHOR RESPONSE

Reviewer: 1

1. This is an excellent well designed, executed and analysed study, with excellent references to comparable studies in other similar countries.
   I noted your replicated about 5% of the interviews. Did you discuss and receive feedback from the interviewers on how thoroughly mothers comprehended the questions and the % where the answers were imprecise enough that no response could be recorded.

Response: Thank you for your comment. We had a quality control plan for the project and 5% reinterview was part of the quality control plan. These interviews were performed by separate data collectors. The data collected by them were compared with the data collected by the study interviewers and if there was >25% mismatch a reinterview was performed.

The comprehensibility of the answers was tested during the pilot testing of the questionnaires and the issues of comprehensibility were resolved at that stage. We did not make changes to the questionnaires once we started the data collection. According to our experience, the respondents could understand and answer these questions.

Accordingly, we did not identify any major discrepancies related to mothers’ understanding of the questions or any instances where responses were too unclear or imprecise to be recorded. During the weekly team meetings with the data collection team, we also discussed the interview process and potential challenges with the interviewers, and no major concerns were reported regarding participants’ comprehension of the questions. Though there were other challenges

related to change in weather, household located in hard-to-reach areas, data collection during festival and ramadan days etc.

2. Could you please summarise your recommendations for specific quantities or weights of foods that need increase in the childrens' diets and enclose your recommendations in a box to highlight them. Also can you provide the costs/pound weight of food needing increases so that the feasibility compared to household income can be assessed.

Response: Thank you for your comment. In this study, we assessed dietary diversity among children using a list-based 24-hour dietary recall approach, adapted from the food group–based dietary diversity assessment method. A list of 24 food categories consumed by children during the 24 hours preceding the interview was collected to assess food group consumption and dietary diversity. However, we did not collect data on portion sizes, quantities, or weights of foods consumed. Therefore, providing recommendations on specific amounts of foods or estimating associated costs is beyond the scope of this study.

Reviewer: 2

1. The aim of this study was to assess the inadequate dietary diversity (IDD) and factors associated with it among 5-9 years old children in Bangladesh. It is very important to address IDD among children. To help improve the manuscript, please consider the following suggestions:

Response: Thank you for your positive comment. We have carefully addressed your suggestions.

2. The authors mentioned that the weighted prevalence of IDD among 5-9 years old children was 52.7%, and the prevalence was higher in boys (55.0%) than in girls (53.2%). However, these differences are small, at only 2.3%, and have not been adjusted for other factors. Therefore, please soften this expression somewhat.

Response: Thank you for your observation. We have revised this expression in the manuscript by changing "higher" to "slightly higher" to reflect the small difference between boys and girls. The changes have been made in the manuscript (page 2, line 43; page 9, line 213).

3. In Bangladesh, school meals primarily in public primary schools in impoverished areas, are provided with the support of NGOs and JICA. These meals consist of a curry-flavored porridge called "khichri," made with soybeans, vegetables, and rice, as well as nutritious bread. If children ate these meals, it would impact IDD. If possible, please provide information on whether any areas provide these school meals. Also, please discuss this in discussion part.

Response: Thank you for raising this important point. We agree that school meals may play an important role in improving dietary diversity among children in this age group. However, information on school meal availability was not collected in our study. We will consider including this variable in future studies. We have added information on school meals on page 2, line 62; page 15, lines 346–348; and page 15, lines 357–359.

VERSION 2 - REVIEW

**Reviewer**	**1**
**Name**	**Thomas, Roger**
**Affiliation**	**University of Calgary, Family Medicine**
**Date**	**11-Jul-2026**
**COI**	

Thank you for the changes to the sample selection and power computation sections. Otherwise you made few changes to the manuscript and explained why the limitations of your data constrained further changes to the manuscript.

**Reviewer**	**2**
**Name**	**Nakamura, Tomiyo**
**Affiliation**	**Ryukoku University, Research Institute for Food and Agriculture**
**Date**	**11-Jul-2026**
**COI**	

I think this manuscript has been properly proofread.
